# Three-year comparison of two mix-and-match strategies: enhanced monofocal and trifocal vs enhanced monofocal and trifocal EDOF intraocular lenses

**DOI:** 10.1097/j.jcrs.0000000000001808

**Published:** 2026-02-20

**Authors:** Victor Danzinger, Marcus Lisy, Daniel Schartmüller, Nikolaus Mahnert, Markus Schranz, Claudette Abela-Formanek, Christina Leydolt

**Affiliations:** From the Department of Ophthalmology and Optometry, Medical University of Vienna, Vienna, Austria.

## Abstract

**Purpose:**

To assess and compare 3-year visual and subjective outcomes after mix-and-match implantation of enhanced monofocal and trifocal or trifocal extended depth-of-focus (EDOF) intraocular lenses (IOLs).

**Setting:**

Department of Ophthalmology and Optometry, Medical University of Vienna, Vienna, Austria.

**Design:**

Single-center, prospective, clinical trial.

**Methods:**

50 patients (100 eyes) received bilateral cataract surgery with implantation of the enhanced monofocal Isopure IOL in the dominant eye and either the trifocal Finevision HP or trifocal EDOF Finevision Triumf IOL in the nondominant eye. At the 3-year examination, binocular uncorrected distance visual acuity (UDVA), uncorrected intermediate visual acuity (UIVA) and uncorrected near visual acuity (UNVA), binocular defocus curves, halo/glare simulation, Catquest-9SF questionnaire, spectacle dependence, and Nd:YAG rate were evaluated.

**Results:**

34 patients (68 eyes) were available at the 3-year follow-up examination. In the Isopure-HP group, binocular UDVA, UIVA (80 cm), UIVA (66 cm), and UNVA were −0.04 ± 0.10 (20/20) logMAR, 0.06 ± 0.07 (20/25) logMAR, 0.10 ± 0.08 (20/25) logMAR, and 0.17 ± 0.08 (20/32) logMAR, respectively. Corresponding values in the Isopure-Triumf group were 0.03 ± 0.11 (20/20) logMAR (*P* = .08), 0.09 ± 0.10 (20/25) logMAR (*P* = .40), 0.12 ± 0.11 (20/25) logMAR (*P* = .56), and 0.27 ± 0.07 (20/40) (*P* < .001). *P* values refer to the difference between both groups. Binocular defocus curves showed ≤0.2 logMAR visual acuities up to −3.0 diopters (D) (Isopure-HP) and −2.0 D (Isopure-Triumf). Halo, glare, and Catquest-9SF scores were comparable (*P* > .05). Nd:YAG treatment was required in 15 (44%) of Isopure eyes, 2 (13%) of Finevision HP eyes, and 1 (6%) of Finevision Triumf eyes.

**Conclusions:**

Combined implantation of enhanced monofocal and trifocal/trifocal EDOF IOLs yields excellent distance and intermediate vision. Near visual acuity and spectacle independence was higher in the Isopure-HP group. Low incidence of photic phenomena and high patient satisfaction highlight the long-term effectiveness of both mix-and-match strategies.

## Abstract 2

This study assessed 3-year outcomes after mix-and-match implantation of enhanced monofocal with trifocal or trifocal EDOF IOLs. Both strategies showed long-term effectiveness, high patient satisfaction, and low photic phenomena.

Traditional monofocal intraocular lenses (IOLs) provide excellent visual acuity at 1 predetermined distance, requiring patients to use spectacles for additional ranges.^1^ Consequently, presbyopia-correcting IOLs were introduced, with multifocal and extended depth-of-focus (EDOF) designs as 2 principal categories.^2^ Multifocal IOLs (mIOLs) create multiple focal points to provide a full range of vision; however, photic phenomena and lower contrast sensitivity are potential disadvantages.^1,3^ Still, near vision and spectacle independence were observed to be highest with mIOLs.^4^ EDOF IOLs incorporate different optical concepts including refractive or diffractive designs with low add power to elongate the depth of focus, providing useful intermediate and distance visual acuity and improved visual quality with a reduced risk of dysphotopsia.^4,5^ A relatively newer group of IOLs, so-called enhanced monofocal IOLs, aim to increase higher-order spherical aberrations for maintained distance and improved intermediate vision, with a comparable risk profile with conventional monofocal IOLs.^6,7^

The combined use of different designs of presbyopia-correcting IOLs, also known as mix-and-match strategy, may compensate for the limitations of a single-lens type while increasing the range of field and spectacle independence.^8–12^ Through binocular summation, this approach may allow patients to gain the additive benefit of both IOL optics, mitigating the disadvantages of a single-lens design.^10,13^ Studies have shown that combining trifocal and EDOF IOLs can increase intermediate and near vision to provide high patient satisfaction and minimal visual disturbance.^8,10^ Similarly, 4 to 6 month results after combined enhanced monofocal and diffractive trifocal or trifocal EDOF IOLs at our department showed good visual acuity results with low values of photic phenomena.^11,12^

Although existing literature reports mainly short-term outcomes, this trial investigates 3-year long-term outcomes after mix-and-match implantation of enhanced monofocal and trifocal or trifocal EDOF IOLs. The aim of this study was to assess and compare long-term objective and subjective clinical results after combined implantation of the enhanced monofocal Isopure IOL in the dominant eye and the trifocal Finevision HP IOL or the trifocal EDOF Finevision Triumf IOL in the nondominant eye (all Physiol SA).

## METHODS

This is an analysis of 3-year outcomes data from a prospective clinical trial that included 2 distinct mix-and-match cohorts at the Department of Ophthalmology and Optometry, Medical University of Vienna, Austria.^11,12^ All participants of the original cohorts (25 patients each, in total 50 patients) were included in this long-term follow-up and were assessed from June 2024 to February 2025. Before study inclusion, all participants provided written informed consent. The study adhered to the tenets of the Declaration of Helsinki and was approved by the local ethics committee of the Medical University of Vienna (EK1379/2024), and trial registration (NCT05573529) was obtained. The inclusion criteria comprised patients anticipated to undergo bilateral cataract surgery with implantation of monofocal enhanced and trifocal or trifocal EDOF IOLs. Mix-and-match implantation was performed only if total corneal astigmatism of each eye was ≤0.75 diopters (D), assessed with the anterior segment optical coherence tomography CASIA2 (Tomey Corp.), and no toric IOL models were used. The analysis dataset included those patients who completed the study-related interventions without intraoperative complications, attended the 3-year follow-up visit, and whose eyes remained free of ocular comorbidities that could affect the visual performance of the IOLs. Exclusion criteria included previous intraocular surgery or ocular trauma, uncontrolled systemic or ocular disease such as uveitis, retinal degenerations or glaucoma, as well as prior refractive laser treatment.

### Surgical Method

All cataract surgeries were performed 3 years prior by 3 expert surgeons (C.L., D.S., and R.M.) using the same surgical protocol through a 2.2 or 2.4 mm clear corneal incision using the Lumera 700 (Carl Zeiss Meditec AG) surgical microscope. Preoperative biometric data were acquired with the IOL Master 700 (Carl Zeiss Meditec AG), and the Barrett Universal II formula was used for IOL power calculations to target emmetropia in all cases.

### The IOLs

One type of enhanced monofocal IOL, the Isopure 123 (Physiol SA), was implanted in the dominant eye. This refractive, aspheric IOL uses a polynomial surface design for increased depth of focus while providing minimal photic phenomena and is made of hydrophobic acrylic material with 4 closed-loop haptics.^14^ In the nondominant eye, either the trifocal FineVision HP POD F GF or the trifocal EDOF FineVision Triumf POD L GF IOL (both Physiol SA) was implanted in 2 consecutive groups to reduce potential dysphotopsia and improve the range of vision. The FineVision HP is an aspheric, apodized diffractive IOL providing +1.75 D intermediate and +3.50 D near add power.^15^ The FineVision Triumf is also an aspheric, diffractive IOL with the same add powers, featuring a modified energy distribution to allocate most light energy to far, less to intermediate, and least to near vision, creating an elongated depth of focus.^16^ Both IOLs use the hydrophobic acrylic material with double–C-loop haptics.

### Three-Year Outcomes

A comprehensive 3-year examination was conducted in all cases. Demographic data, date of the surgery, lens type, and power were recorded. Subjective refraction including sphere, cylinder, and axis were evaluated in photopic light conditions by 2 experienced optometrists masked to the type of IOLs implanted in each eye. Uncorrected binocular defocus curves (+1.0 to −4.0 D) and uncorrected binocular visual acuities for distance (4 m), intermediate (80 cm and 66 cm), and near (40 cm) distances were evaluated on corresponding Early Treatment Diabetic Retinopathy Study (ETDRS) charts (Good-Lite Company/Precision Vision) in photopic conditions. The study charts were changed periodically to avoid any learning bias and visual acuities were measured using the logMAR scale. Photic phenomena were tested using a virtual Halo and Glare simulator (Eyeland Design Network GmbH). Patients were instructed to select and modify for halo/glare size and intensity on a slide bar ranging from 0 (minimum) to 100 (maximum) on simulated night-time driving. The Catquest-9SF 9-item questionnaire was administered to assess perceived difficulty in daily-life activities (1 = no difficulty to 4 = great difficulty) as well as overall visual satisfaction (1 = very satisfied to 4 = very dissatisfied). The ordinal raw data were then converted to Rasch-adjusted scores (logits), as previously described by the authors of the questionnaire to account for varying item difficulty and unequal distance between response options, enabling parametric statistical analysis.^17^ Higher (positive or closer to 0) Rasch scores indicate greater difficulty with tasks and lower satisfaction, with total Rash-calibrated person scores ranging from min: −3.03 logits to max: 2.64 logits.^17^ Spectacle-dependence (1 = never, 2 = sometimes, 3 = always) was evaluated at far, intermediate, and near distance. Exploratory assessment included a slitlamp examination to exclude severe posterior capsular opacification (PCO), and the number of previous Nd:YAG capsulotomies was recorded. In the case of clinically significant central PCO and visual acuity of ≤0.8 Snellen, Nd:YAG treatment was performed, and study assessment was postponed for 2 to 3 weeks. In addition, intraocular pressure measurements and fundus examination were performed to rule out any further ophthalmic diseases such as glaucoma, uveitis, or retinal pathologies.

### Objectives

The primary objective was to compare binocular uncorrected near visual acuity (UNVA) 3 years after combined enhanced monofocal and trifocal IOL (Group 1: Isopure-HP) or trifocal EDOF IOL (Group 2: Isopure-Triumf) implantation. Further visual and subjective outcomes serve as secondary results.

### Statistics and Sample Size

No new a priori sample size calculation was performed for this 3-year follow-up because the trial aimed to assess and compare the uncorrected binocular outcomes of 2 existing cohorts.^11,12^ However, a post hoc power analysis was performed to evaluate the sufficiency of the sample size. To detect a difference of 1 logMAR line between 2 groups, assuming 0.1 SD, 80% power, and alpha 0.05, a sample size of 16 patients per group is required. Therefore, our 2 cohorts of 16 and 18 patients are sufficiently powered to compare visual outcome parameters.

For the analysis of the primary objective, descriptive statistics were performed to obtain mean values, SD, and range (minimum, maximum). Descriptive statistics and frequencies were used in categorical variables. The normality of the data was analyzed using the Shapiro-Wilk test. The independent *t* test was applied to compare mean values in normally distributed data, and the Mann-Whitney *U* test was used in nonnormally distributed data. *P* values of secondary outcomes were exploratory and serve for descriptive purposes; therefore, no multiplicity corrections were applied. All tests were performed 2-tailed, and the level of statistical significance was set at *P* < .05. Data analysis was performed using IBM SPSS v. 29.0.2.0 (SPSS, Inc.) and Microsoft Excel for Mac v. 16.35 (Microsoft Corp.).

## RESULTS

### Patient Demographics and Refractive Outcomes

**Figure 1.**
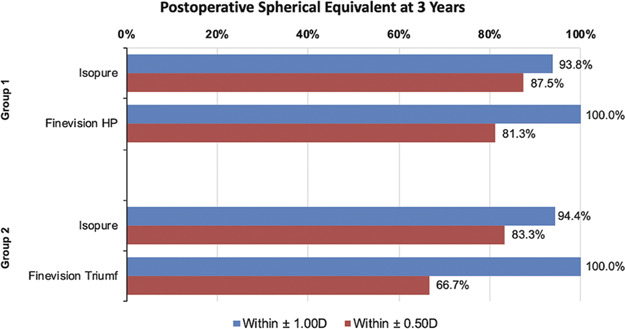
Postoperative spherical equivalent 3 years after mix-and-match implantation of the enhanced monofocal Isopure IOL and the trifocal Finevision HP IOL (Group 1) or the trifocal EDOF Finevision Triumf IOL (Group 2).

Of the 50 patients who previously underwent combined enhanced monofocal and trifocal or trifocal EDOF IOL implantation at our department, 34 (68%) patients were available and consented to the 3-year follow-up examination. Reasons for exclusion were unable to contact (7), no-show for follow-up (2), refusal to participate (1), death during the follow-up period (2), or development of conditions affecting visual outcome such as epiretinal membrane (2), anterior uveitis unrelated to the IOLs (1), or corneal guttata (1). In the final study cohort, there were 16 participants in the Isopure-HP group and 18 participants in the Isopure-Triumf group. Further patient characteristics are displayed in Table 1. In both mix-and-match groups, there were no significant differences in sphere, cylinder, or postoperative spherical equivalent (SEQ) between corresponding enhanced monofocal eyes or trifocal and trifocal EDOF eyes (Table 1). Refractive predictability was high in both groups: For the Isopure-HP group, 93.8% (15/16) of Isopure eyes and 100% (16/16) of Finevision HP eyes achieved ±1.00 D SEQ. Similarly, the Isopure-Triumf group showed 94.4% (17/18) of Isopure eyes and 100% (18/18) of Finevision Triumf eyes within this range. Regarding ±0.50 D SEQ, the first group achieved 87.5% (14/16) and 81.3% (13/16) in Isopure and Finevision HP eyes, respectively. The second group demonstrated 83.3% (15/18) of Isopure eyes and 66.7% (12/18) of Finevision Triumf eyes within ±0.50 D SEQ (Figure 1).

**Table 1.** Patient demographics and refractive outcomes for the Isopure-HP group and the Isopure-Triumf group at 3 years

Parameter	Isopure-HP (Group 1)		Isopure-Triumf (Group 2)		*P* value^a^
Patients (eyes)	16 (32)		18 (36)		
Sex (M/F)	5/11		3/15		
Age (y)	71.5 ± 9.0 (59.2, 85.1)		76.8 ± 5.7 (67.8, 88.2)		
Surgery to follow-up (y)	3.2 ± 0.3 (2.6, 3.6)		2.6 ± 0.3 (2.1, 3.2)		
IOL	Isopure	Finevision HP	Isopure	Finevision Triumf	
	A	B	C	D	
IOL power (D)	23.84 ± 2.05 (19.50, 27.50)	23.81 ± 2.38 (19.00, 28.50)	21.86 ± 2.44 (16.00, 26.00)	21.50 ± 2.81 (14.50, 25.50)	
Sphere (D)	−0.11 ± 0.46 (−0.75, 1.00)	−0.19 ± 0.44 (−1.00, 0.50)	−0.25 ± 0.38 (−1.25, 0.25)	−0.29 ± 0.38 (−1.00, 0.25)	A-C .56 B-D .79
Cylinder (D)	0.27 ± 0.32 (0.00, 1.00)	0.42 ± 0.43 (0.00, 1.50)	0.18 ± 0.23 (0.00, 0.75)	0.22 ± 0.22 (0.00, 0.75)	A-C .55 B-D .24
SEQ	0.02 ± 0.45 (−0.63, 1.25)	0.02 ± 0.41 (−0.63, 1.00)	−0.16 ± 0.40 (−1.25, 0.50)	−0.27 ± 0.39 (−0.88, 0.38)	A-C .44 B-D .12

a Mann-Whitney *U* test used for comparison

### Three-Year Visual Outcomes

**Figure 2.**
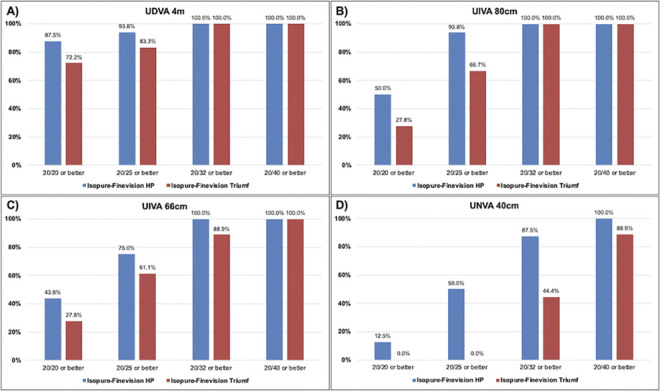
Cumulative binocular uncorrected visual acuity at (*A*) 4 m distance (UDVA), (*B*) 80 cm intermediate (UIVA 80 cm), (*C*) 66 cm intermediate (UIVA 66 cm), and (*D*) 40 cm near (UNVA 40 cm) distance for the Isopure-HP group and the Isopure-Triumf group at 3 years.

Binocular uncorrected visual acuities at far, intermediate, and near distance are displayed in Table 2. Binocular uncorrected distance visual acuity (UDVA) at 4 m distance was close to 0.0 logMAR (20/20 Snellen) in both the Isopure-HP (−0.04 ± 0.10 logMAR) and Isopure-Triumf (0.03 ± 0.11 logMAR) groups, with an almost statistically significant difference (*P* = .08). Similar results were observed for binocular uncorrected intermediate visual acuity (UIVA) at 80 cm (*P* = .40) and 66 cm (*P* = .56) and were close or equivalent to 0.1 logMAR (20/25 Snellen) in both mix-and-match groups. However, binocular UNVA at 40 cm was higher in the Isopure-HP group, demonstrating statistically significant (*P* < .001) and clinically significant differences of 0.1 logMAR corresponding to 5 ETDRS letters or 1-line difference on the ETDRS study chart. The distribution of cumulative visual acuity results of both mix-and-match groups is displayed in Figure 2. Binocular UDVA was ≥20/25 Snellen in 93.8% and 83.3% of patients in the Isopure-HP and Isopure-Triumf groups. Similarly, binocular UIVA at 80 cm was ≥20/25 Snellen in 93.8% and 66.7%, binocular UIVA at 66 cm was ≥20/25 Snellen in 75.0% and 61.1%, and binocular UNVA at 40 cm was ≥20/32 Snellen in 87.5% and 44.4% of patients for the respective groups.

**Table 2.** Binocular uncorrected visual outcomes for the Isopure-HP group and the Isopure-Triumf group at 3 years

VA (logMAR)	Isopure-HP (Group 1) Mean ± SD (range)	Isopure-Triumf (Group 2) Mean ± SD (range)	*P* value^a^
UDVA at 4 m	−0.04 ± 0.10 (−0.20, 0.16)	0.03 ± 0.11 (−0.16, 0.24)	.08
UIVA at 80 cm	0.06 ± 0.07 (−0.08, 0.20)	0.09 ± 0.10 (−0.10, 0.20)	.40
UIVA at 66 cm	0.10 ± 0.08 (0.00, 0.24)	0.12 ± 0.11 (−0.10, 0.30)	.56
UNVA at 40 cm	0.17 ± 0.08 (0.02, 0.34)	0.27 ± 0.07 (0.16, 0.40)	<.001*

* Statistically significant

a Independent *t* test used for comparison

**Figure 3.**
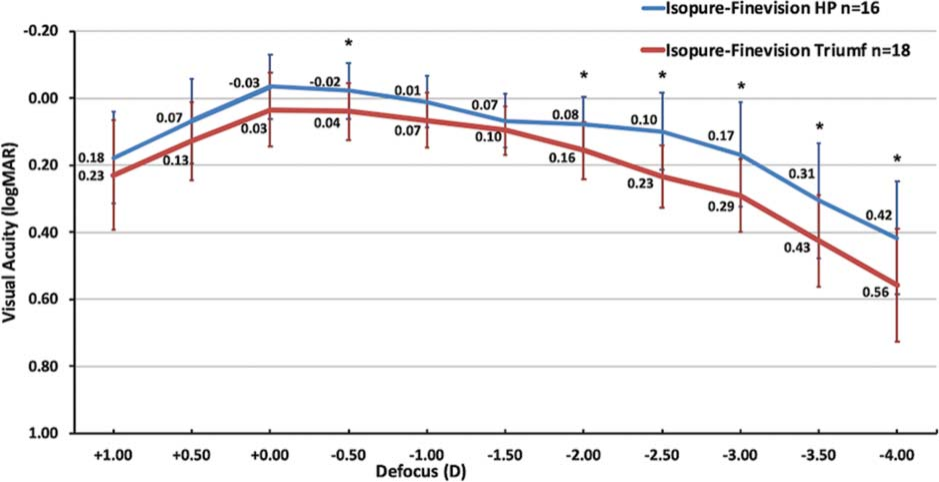
Binocular uncorrected defocus curves of the Isopure-HP group and the Isopure-Triumf group at 3 years. *P < .05.

Uncorrected binocular defocus curves exhibited a full range of functional vision from near to distance in both mix-and-match groups (Figure 3). The Isopure-HP group demonstrated a wider curve with visual acuities of 0.2 logMAR (Snellen 20/32) or better between +1.00 and −3.00 D levels of defocus compared with +0.50 D and −2.00 D in the Isopure-Triumf group. Peak distance visual acuities were observed at 0.0 D, with a trend of increased visual performance in the Isopure-HP group almost achieving statistical significance (*P* = .07). Further statistically significant differences were detected at −0.50 D (*P* = .048), −2.00 D (*P* = .01), −2.50 D (*P* < .001), −3.00 D (*P* = .02), −3.50 D (*P* = .04), and −4.00 D (*P* = .03) levels of defocus.

### Patient-Reported Outcomes

Rasch-calibrated results of the Catquest-9SF questionnaire and spectacle dependence at various distances are displayed within Supplemental Table 1 (available at http://links.lww.com/JRS/B496). Spectacle independence at distance was high (94%) and comparable in both groups (*P* = .90). Although not statistically significant, a trend in spectacle independence favoring the Isopure-HP group was observed at near distance (Isopure-HP: 50% vs Isopure-Triumf: 22%, *P* = .14) and for any purpose at all distances (Isopure-HP: 50% vs Isopure-Triumf: 17%, *P* = .09). The mean postoperative Rasch-calibrated Catquest-9SF person score was −2.89 ± 0.25 logits (−3.03 to −2.10) in the Isopure-HP group and −2.88 ± 0.21 logits (−3.03 to −2.32) in the Isopure-Triumf group (*P* = .62). There were no statistically significant differences between both mix-and-match groups for any survey item observed.

### Photic Phenomena

**Figure 4.**
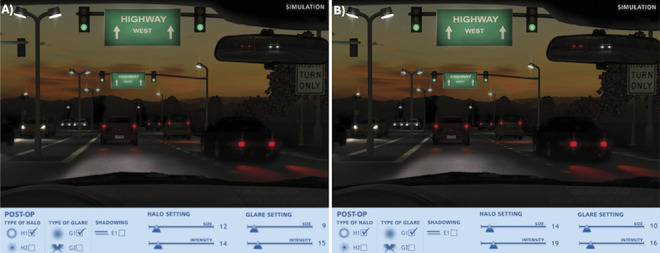
Halo and glare mean values of perceived photic phenomena for (*A*) the Isopure-HP group and (*B*) the Isopure-Triumf group at 3 years.

Using the virtual Halo and Glare simulator, overall low levels of photic phenomena were observed in both mix-and-match groups, corresponding mean values are displayed in Figure 4, respectively. The Isopure-HP and Isopure-Triumf groups revealed similar halo size (11.5 ± 14.4 vs 14.3 ± 12.5; *P* = .27) and intensity (13.8 ± 18.0 vs 18.8 ± 16.0; *P* = .22). Accordingly, no significant differences were observed in glare size (8.8 ± 10.9 vs 10.4 ± 6.9; *P* = .18) or intensity (14.9 ± 14.3 vs 15.7 ± 10.9; *P* = .76) between the respective groups.

### Exploratory Outcomes

At 3 years, 15 (44%) of the 34 eyes implanted with the Isopure IOL, 2 (13%) of the 16 eyes with the Finevision HP IOL, and 1 (6%) of the 18 eyes with the Finevision Triumf IOL experienced a clinically significant PCO requiring Nd:YAG treatment. In cases receiving treatment at our department, study assessments were postponed by a 2- to 3-week interval.

## DISCUSSION

In this 3-year follow-up study, we report long-term refractive, visual, and patient-reported outcomes of patients receiving combined enhanced monofocal and trifocal/trifocal EDOF IOL implantation. Both the Isopure-HP and Isopure-Triumf groups exhibited excellent mean binocular UDVA of −0.04 ± 0.10 logMAR and 0.03 ± 0.11 logMAR, respectively, with an almost statistically significant difference (*P* = .08). In total, a high percentage of patients achieved 20/25 Snellen or better (93.8% vs 83.3%) at distance (Table 2, Figure 2A). Similarly, both groups showed high visual outcomes at intermediate distance close to 0.1 logMAR at 80 cm UIVA (*P* = .40) and 66 cm UIVA (*P* = .56) (Table 2). Accordingly, a significant proportion of patients achieved 20/25 Snellen or better at 80 cm (93.8% vs 66.7%) and 66 cm (75.0% vs 61.1%) distance (Figure 2B and C). At 40 cm near distance, the Isopure-HP group showed significantly better binocular UNVA compared with the Isopure-Triumf group (0.17 ± 0.08 vs 0.27 ± 0.07 logMAR, *P* < .001) (Table 2). Notably, 87.5% in the first group were able to read 20/32 Snellen or better, compared with less than half (44.4%) in the second group (Figure 2D). Binocular uncorrected defocus curves confirm these observations, demonstrating an overall broader curve in the Isopure-HP group, with a trend of better peak visual performance at 0.0 D (*P* = .07) (Figure 3). Accordingly, statistically significant differences were detected at defocus levels simulating near vision between −2.00 D and −4.00 D (all *P* < .05). As there were no statistically significant differences of the postoperative SEQ, outcome differences are likely attributed to the different light energy distributions of the mIOLs, with the Finevision Triumf IOL prioritizing intermediate over near vision, as previously demonstrated in optical bench analysis by Labuz et al. (Table 1).^18^ Notably, although the standard for defocus curve testing involves correcting for residual refractive errors to allow for a direct comparison of IOL optics as highlighted by Cionni et al., our study design was an uncorrected approach to evaluate the real-world, functional vision of both our mix-and-match groups.^19^ As noted by Kohnen et al., while monocular defocus testing is essential to assess an IOL's intrinsic optical performance, binocular defocus testing generally exhibits better visual outcome.^20^ However, binocular examination may simulate vision during everyday life more accurately than monocular defocus testing, particularly relevant in mix-and-match strategies.^21^ Thus, our uncorrected binocular approach reflects the clinical measure of patient visual outcome.

Correspondingly, previously published short-term results by our group observed lower monocular distance-corrected near visual acuities and a larger difference between the Finevision HP and the Finevision Triumf IOL (distance-corrected near visual acuity HP: 0.20 ± 0.15, Triumf: 0.31 ± 0.10), and improved uncorrected values in this study are presumably due to binocular summation achieved through the mix-and-match approach (Table 2).^11,12^ Similarly, monocular defocus curves of our 2 short-term trials showed a typical bimodal curve of the Finevision HP IOL, whereas the Finevision Triumf IOL exhibited a relatively flat curve.^11,12^ Accordingly, a study by Kim et al. reported monocular distance-corrected defocus curves of both these IOLs, showing a significant decrease for the Finevision Triumf IOL between −2.00 and −4.00 D compared with the Finevision HP and other trifocal IOLs, whereas, in contrast to our findings, a smaller difference in monocular UNVA was reported (UNVA HP: 0.04 ± 0.06, Triumf: 0.09 ± 0.09).^22^

Compared with bilateral enhanced monofocal Isopure IOL implantation, both mix-and-match strategies of this trial demonstrated comparable binocular UDVA.^23–26^ Moreover, binocular UIVA was similar at 80 cm or 1 line (0.1 logMAR or 5 EDTRS letters) better at 66 cm compared with these previous reports.^23–26^ Only 1 Mini-Monovision trial using the enhanced monofocal Isopure IOL–reported binocular UNVA of 0.40 ± 0.20 logMAR at 33 cm, which was 1 (Isopure-HP) to 2 (Isopure-Triumf) lines lower compared with our mix-and-match results obtained at 40 cm distance (Table 2).^26^

In addition to the assessment of long-term clinical outcomes, the well-established and validated Catquest-9SF questionnaire was applied to evaluate patient-reported outcome.^17^ In both groups, Rasch-calibrated person scores indicated low difficulties during daily-life activities and high patient satisfaction because more negative logit scores signify a better subjective visual function. The mean Rasch person score was −2.89 ± 0.25 logits in the Isopure-HP group and −2.88 ± 0.21 logits in the Isopure-Triumf group, showing no significant difference (*P* = .62) between both groups (Supplemental Table 1, available at http://links.lww.com/JRS/B496). Spectacle dependence varied by distance in both groups: At far distance, high spectacle independence was observed in both groups (94%). For intermediate vision, all patients (100%) in the Finevision-Triumf group reported spectacle independence compared with 88% in the Isopure-HP group, presumably due to the higher energy split at intermediate distance of the Triumf IOL. At near vision, partial spectacle independence (“sometimes”) was lower in the Isopure-HP group (44%) compared with the Isopure-Triumf (72%) but notably higher for complete spectacle independence (“never”) using the Isopure-HP approach (50%) compared with the Finevision-Triumf (17%), although the total difference was not statistically significant (*P* = .14) (Supplemental Table 1, available at http://links.lww.com/JRS/B496). Furthermore, low and comparable values of photic phenomena were observed in both groups and highlight the efficacy of both mix-and-match strategies (Figure 4). By implanting the enhanced monofocal Isopure IOL in the dominant eye, we aimed for good distance vision while enhancing intermediate vision and providing minimal photic disturbances. Simultaneously, the trifocal Finevision HP or trifocal EDOF Triumf IOL was implanted in the nondominant eye to increase near vision. Notably, halo and glare results were comparable with our 2 short-term trials and with the findings of Ang et al. evaluating bilateral implantation of the Isopure IOL.^11,12,23^

The limitations of this trial were the absence of monocular or distance-corrected visual acuities because these data were reported previously in our short-term studies.^11,12^ Furthermore, this study assessed uncorrected binocular defocus curves, and residual refractive errors may have influenced outcome variability. However, this trial specifically aimed to evaluate long-term, clinical data and patient satisfaction of 2 consecutive mix-and-match groups. Moreover, a prospective randomized design including a priori sample size calculation would have strengthened the generalizability of our results. Further limitations include the lack of contrast sensitivity evaluation at different spatial frequencies and the absence of pupil size measurements. The strengths of this trial were the long follow-up period, and a total of 68% of previously operated patients at our department were analyzed after 3 years. Furthermore, visual outcome was assessed systematically with masking of the assessing optometrists to the type of IOL implanted.

To conclude, this study reports excellent visual performance and patient satisfaction 3 years after mix-and-match implantation of an enhanced monofocal IOL in the dominant eye and a trifocal or trifocal EDOF IOL in the nondominant eye. The results suggest that combined implantation of these types of IOLs is a promising treatment option to increase the range of vision providing high spectacle independence while reducing dysphotopsia long-term.

**WHAT WAS KNOWN**

- Enhanced monofocal IOLs provide excellent distance vision with improved intermediate vision.
- Multifocal IOLs enhance near vision but may induce disturbing photic phenomena.
- Mix-and-match strategies aim to combine various IOL designs to optimize visual outcomes while minimizing potential disadvantages.

**WHAT THIS PAPER ADDS**

- This study reports the long-term effectiveness of two mix-and-match strategies combining enhanced monofocal with trifocal or trifocal EDOF IOLs.
- At 3-years, both combinations demonstrated a broad range of vision, low photic phenomena and high patient satisfaction, confirming their long-term tolerability.
